# Interactions between the oyster larvae pathogen *Vibrio ostreicida* and the bivalve hosts *Mytilus galloprovincialis* and *Magallana gigas*

**DOI:** 10.3389/fimmu.2025.1711477

**Published:** 2025-11-26

**Authors:** Martina Leonessi, Manon Auguste, Jose R. Lopez, Teresa Balbi, Caterina Ciacci, Caterina Oliveri, Luigi Vezzulli, Dolors Furones, Laura Canesi

**Affiliations:** 1 Department of Earth, Environmental and Life Sciences (DISTAV), University of Genoa, Genoa, Italy; 2 National Biodiversity Future Center, Palermo, Italy; 3 Aquaculture Program, Institute of Agrifood Research and Technology (IRTA), La Ràpita, Spain; 4 Universidade de Santiago de Compostela, Santiago de Compostela, Spain; 5 Department of Biomolecular Sciences (DIBS), University ‘Carlo Bo’ of Urbino, Urbino, Italy

**Keywords:** bivalves, *Vibrio ostreicida*, larvae, hemocytes, immune response, mussel, oyster

## Abstract

**Introduction:**

Marine bivalves are continuously exposed to a variety of environmental stressors, including different Vibrio species potentially involved in disease outbreaks that have severely impacted farmed populations over the past two decades. *Vibrio ostreicida* was firstly identified as a lethal pathogen for oyster larvae (*Ostrea edulis*), but its interactions with the immune system of the bivalve host are largely unexplored.

**Methods:**

In this study, we investigated the pathogenic potential of a *V. ostreicida* strain r172, isolated from a 2022 mortality event in adult *Mytilus galloprovincialis* in the Ebro Delta (Spain), focusing on mussel early larval development and hemolymph immune responses in *in vitro* short-term exposure experiments. Immune responses were compared with those of the oyster *Magallana gigas*.

**Results:**

Both live and heat-killed *V. ostreicida* significantly impaired normal larval development with a dose-concentration effect (EC_50_ ≈ 10^3^ - 10_4_ CFU/mL), with live bacteria inducing shell malformations and heat-killed Vibrio causing developmental arrest. In the hemocytes of adult mussels, exposure to heat-killed *V. ostreicida* led to dose-dependent lysosomal destabilization, reduced phagocytic activity, and increased intracellular ROS production. Similar lysosomal destabilization was observed with live *V. ostreicida*, which also stimulated extracellular ROS and nitric oxide release, but only in the presence of hemolymph serum (HS). Mussel HS displayed a strong bactericidal activity towards *V. ostreicida*, highlighting a key role for soluble immune effectors. In oyster hemocytes, *V. ostreicida* induced similar lysosomal stress; however, neither hemocytes nor serum showed any bactericidal activity towards this strain.

**Discussion:**

This data represents the first attempt to elucidate the mechanisms underlying the interactions of an environmental strain of *V. ostreicida* with marine bivalves. The species-specific differences observed in immune responses highlight the complexity of host-pathogen interactions in these organisms and emphasize the need for further investigation into immune responses of different aquacultured species.

## 1 Introduction

In the last decade, recurrent disease and mass mortality events of both farmed and natural stocks of marine bivalves have been observed throughout Europe. Even though the exact causes are not always clear, and these events are generally considered both multifactorial and polymicrobial, bacteria, in particular members of the genus *Vibrio*, have been identified as the main infectious agents in different bivalve species (1–6).

Among bivalves, oysters have been shown to be historically more affected, with several Vibrio species identified as main pathogens. In particular, members of the Splendidus clade (*V.* sp*lendidus*, *V. tasmaniensis*, and *V. crassostreae*) and *V. aestuarianus*, have been associated with disease outbreaks across France, Ireland (7) and Spain, notably in the Ebro Delta in 2016–2019 (8). Several vibrio isolates were confirmed as virulent in experimental infections (9–13). Vibrioses have been also described in other bivalve species, such as those caused by *V. tubiashii* in the scallop *Argopecten irradians*, or by *V. tapetis*, the causative agent of the well-known Brown Ring Disease in *Ruditapes philippinarum* (1, 2).

Some vibrios have been also implicated in larval mortalities across different host species, such as *V. tubiashii* in the oysters *Magallana gigas* and *Ostrea edulis*, and in the scallop *Argopecten irradians* (2). Also *Mytilus galloprovincialis* early larval stages have been shown to be susceptible to challenge with different vibrios, such as V. *bathipelagicus*, which induced larval death (14), or. *V. coralliilyticus* and *V. tasmaniensis* LGP32 (14, 15) that induced developmental arrest.

On the other hand, adult mussels (*Mytilus* spp.) generally show a remarkable resistance to invading vibrios with respect to other bivalve species, essentially due to the efficiency of their immune system. Differences in immune effectors between oysters and mussels may underlie the generally greater resilience of mussels to bacterial infections (16, 17). However, this advantageous trait seems to be not sufficient to face the more recent threats hitting European coastal waters. Indeed, increasing reports of mortality events in *M. edulis* and *M. galloprovincialis* have been recorded across Europe since 2014 (18–21). The fast changes of environmental variables seemed to be a triggering factor (17, 22); in addition, some bacteria belonging to the *Vibrio* genus, *V.* sp*lendidus* in particular, were also isolated from mussel mortality episodes (6, 22, 23). However, so far, no particular bacterium has been identified as an etiological agent of disease in *Mytilus* spp.

Previous episodes of disease and severe mortalities were observed in young oyster spat (*O. edulis*) in a bivalve hatchery in Galicia (NW Spain) from 1999 (24). Among the bacterial strains isolated from samples of oysters, water tanks, and surface of the affected tanks, one *Vibrio* species induced > 98.5% mortality in virulence tests carried out with oyster larvae (10–12 days old) at 22°C (Prado et al., 2005). The type strain, PP-203T (5CECT 7398T5DSM 21433T), isolated from the inner surface of containers, was subsequently identified as *V. ostreicida* (25), and its draft genome subsequently released in 2020 (26). However, since no further mentions of *V. ostreicida* interactions with oysters or other bivalves have appeared in the literature since then, little is known about its epidemiology, host range or virulence.

We have recently reported the isolation of strains of *V. ostreicida* from a mortality episode occurred in spring 2022 in adult *M. galloprovincialis* farmed in the Ebro River Delta in NW Spain, involving 25% of individuals, but observed at a water temperature of 18 °C, lower than in the other usual summer outbreaks (27). Histological examination revealed bacterial foci and tissue damage compatible with bacterial toxicity. Bacterial isolates from mussel sampled at the affected site showed a distinct profile; the phenotypic characterization of three representative isolates of this profile (r167, r172, r190) allowed their identification as *Vibrio ostreicida* (27). The complete genome of the r172 isolate was sequenced and preliminary virulence experiments in adult mussels showed mortality of 50% at 5 days post infection (27). This data suggested that this *V. ostreicida* strain may also represent a pathogen for mussels. However, to our knowledge, no data is available on the virulence of *V. ostreicida* in adult oysters or other bivalve species.

In this work, responses to the *V. ostreicida* r172 were investigated in *M. galloprovincialis*. *In vivo* experiments were performed in mussel larvae (48 h post-fertilization, hpf), in order to evaluate pathogenicity with early developmental stages. *In vitro*, short term responses of mussel hemolymph components to both heat-killed and live *V. ostreicida* were evaluated in terms of hemocyte lysosomal membrane stability (LMS), phagocytosis and bactericidal activity. Extracellular lysozyme release, Reactive Oxygen Species (ROS) and Nitric oxide (NO) production were also evaluated. The main results (LMS and bactericidal activity) were compared with those obtained with hemolymph components of the oyster *Magallana gigas*.

## 2 Materials and methods

### 2.1 Bacterial cultures and inoculum preparation

The *V. ostreicida* strain *r172* was isolated from an adult mussel mortality episode that occurred in April 2022 in an aquaculture farm in Alfacs Bay, Ebro River Delta (Tarragona, Spain) (27), close to the research center of IRTA- La Ràpita. *V. ostreicida r172* was cultured in Zobell Marine Broth at 23°C under static conditions. After 5–6 h growth, cells in exponential phase were harvested by centrifugation (4,500 x g for 10 min), washed once with filter-sterilized artificial sea water (ASW, 35 ‰ for mussel experiments and 31 ‰ for oysters), and resuspended in ASW or mussel hemolymph serum (HS) to obtain a concentration of about 10^8^ CFU/mL (determined spectrophotometrically as an Abs 600 = 0.38 of a suspension diluted 1:10). Marine agar (MA) was used throughout the experiments. For assays with heat-killed bacteria, samples were boiled for 10 min in a water bath, stored at 4°C and the whole unwashed suspensions suitably diluted in ASW to obtain the desire concentrations. This type of preparation was previously utilized in first *in vitro* studies on mussel hemocytes challenged with the known bivalve pathogen *V. splendidus* LGP32. as previously described (28).

### 2.2 Animals

*Mytilus galloprovincialis*, 4–6 cm long, were purchased from a mussel farm in the Ligurian Sea (La Spezia, Italy) in November 2022 (for larval assays) and in spring-summer 2023 (for immune assays). Animals were transferred to the laboratory and acclimatized in static tanks containing aerated ASW (1 L/animal), pH 7.9-8.1, 35 ‰ at 18°C for 24 h. Mussels were not fed during experiments. Specimens of *Magallana gigas*, 8–10 cm long, were purchased from an aquaculture farm (Bretagne, France) and acclimatized for 24 h in the same conditions, except for lower ASW salinity (31 ‰).

### 2.3 Effects of *V. ostreicida* on mussel early larval development

The mussel 48-h embryotoxicity assay (29) was carried out in 96-microwell plates as previously described (15, 30, 31). At 30 min post-fertilization, aliquots of 20 μL of live and heat-killed *V. ostreicida* suspensions, suitably diluted, were added to fertilized eggs in each microwell to reach the nominal final concentrations (from 10^2^ to 10^8^ CFU/mL) in a 200 μL volume. Control samples in ASW were run in parallel. Microplates were then incubated for 48 h at 18°C ± 1°C, with a 16:8 h light: dark photoperiod. All the procedures were carried out following (29). At the end of incubation, samples were fixed with buffered formalin (4%). A larva was considered normal when the shell was D-shaped (straight hinge) and the mantle did not protrude out of the shell, and malformed if had not reached the stage typical for 48 h (trocophora or earlier stages) or when some developmental defects were observed (concave, malformed or damaged shell, protruding mantle). The recorded endpoint was the percentage of normal D-larvae in each well with respect to the total, including malformed larvae and pre-D stages. The acceptability of test results was based on controls for a percentage of normal D-shell stage larvae >75% (29). Experiments were performed using fertilized eggs obtained by different parental pairs (N = 3), each in 6 replicate wells per condition (see Methods in **Supplementary Information** for details). All larvae in each well were examined by optical microscopy using an inverted Olympus IX53 microscope (Olympus, Milano, Italy) at 400 x, equipped with a CCD UC30 camera and a digital image acquisition software (cellSens Entry).

### 2.4 *In vitro* challenge of *Mytilus galloprovincialis* hemocytes with *V. ostreicida*

#### 2.4.1 Hemolymph sampling and sample preparation

All procedures were performed as previously described (30). Hemolymph was extracted from the posterior adductor muscle of 10–15 mussels using a sterile 1 mL syringe with an 18 G1/2” needle, filtered through sterile gauze and pooled in Falcon tubes at 18°C as previously described. Hemolymph serum (HS) was obtained by centrifugation of whole hemolymph at 100 *x g* for 10 min and the supernatant was sterilized through a 0.22 µm-pore filter. Hemocyte monolayers were prepared by depositing 20 µL drops of whole hemolymph on microscope slides and the cells were left to adhere at 18°C for 20 min before removing the excess hemolymph (30). Hemocyte monolayers or aliquots of 500 µL of whole hemolymph samples (according to the endpoint) were incubated with either live or heat-killed *V. ostreicida* suspensions suitably diluted in ASW or HS at different final concentrations (from 10^8^ to 10^5^ CFU/mL) from 30 to 90 minutes. Untreated samples in ASW or HS were run in parallel. All experiments were carried out at 35 ‰ salinity.

#### 2.4.2 Hemocyte functional parameters

Lysosomal membrane stability (LMS) and phagocytic activity were evaluated in hemocyte monolayers as previously described (15, 30, 32). LMS was determined after 30 min incubation with either heat-killed (in ASW) or live bacteria (resuspended in ASW or HS) or at different concentrations (from 10^5^ to 10^8^ CFU/mL). In all functional assays the bacteria:hemocyte ratio was 3:1 for a bacterial concentration of 10^6^ CFU/mL. This ratio changed accordingly at different vibrio concentrations.

The phagocytic activity was evaluated by the uptake of NR-stained zymosan incubated for 60 min with only heat-killed bacteria (from 10^6^ to 10^8^ CFU/mL).This experimental choice was made because some bacteria are known to evade the host immune defense by interfering with phagocytic process of the host cells (33). Therefore, in the presence of live bacteria, with this method it cannot be identified whether a change in particle uptake may reflect a preference of hemocytes towards *V. ostreicida* with respect to zymosan (or vice versa) or interference with the phagocytic process by live vibrios.

Lysozyme activity, extracellular reactive oxygen species (ROS) and nitric oxide (NO) production were determined in whole hemolymph samples as previously described (15, 30, 32). See more details in **Supplementary Information** (Methods).

The effects of heat-killed *V. ostreicida* (10^6^ and 10^7^ CFU/mL, 30 min) were further investigated by confocal fluorescence microscopy in hemocytes loaded with LysoTracker Green (LTG) (250 nM, 30 min) (Thermo Fisher Scientific, Massachusetts, USA) and 2’,7’-Dichlorofluorescein (DCF) (2 µM, 30 min) (Sigma, Milan, Italy) as previously described (34). LTG is a green, fluorescent dye that stains acidic compartments in live cells. DCF fluorescence is used as an indicator for production reactive oxygen species (ROS) in live cells. Fluorescence of Lysotracker Green (ex: 504 nm, em: 511 nm) and DCF (ex: 495 nm, em: 520 nm) were detected using a Leica TCS SP5 confocal setup mounted on a Leica DMI 6000 CS inverted microscope (Leica Microsystems, Heidelberg, Germany) using 63 ×1.4 oil objective (HCX PL APO ×63.0 1.40 OIL UV). Images were analyzed by the Leica Application Suite Advanced Fluorescence (LASAF) and ImageJ Software (Wayne Rasband, Bethesda, MA).

Bactericidal activity of hemocytes towards live *V. ostreicida* was evaluated as previously described (30). Briefly, hemocyte monolayers (obtained from 500 µL hemolymph) were incubated in 24 multi-well plates with live *V. ostreicida* (5 x 10^6^ CFU/mL) at 18 °C in ASW or HS for different periods of time (0, 30 and 90 min). The bactericidal activity of HS alone (aliquots of 500 µL) was also evaluated. *V. ostreicida* in ASW was used as a control for bacterial growth over the experimental time. Immediately after the inoculum (time 0) and after 30 and 90 min of incubation, supernatants were collected and hemocytes were lysed by adding filter-sterilized ASW with 0.05% Triton x-100 and by 10 s agitation. Supernatants and lysates were pooled and tenfold serial diluted in ASW. Aliquots of 100 µL of the diluted samples were plated onto MA. Samples of *V. ostreicida* resuspended in ASW or HS at the same concentrations were directly diluted and plated. After 24 h incubation at 23°C, the number of CFU per hemocyte monolayer was determined. Percentages of killing were determined in comparison to values obtained at time zero. The number of CFU in control hemocytes never exceeded 0.1% of those counted in vibrio-exposed samples. In all experiments, ASW at 35 ‰ salinity was utilized.

### 2.5 *In vitro* challenge of *Magallana gigas* hemocytes with *V. ostreicida*

Hemolymph sampling and monolayer preparations were performed as described above with slight modifications (14). Effects of heat-killed and live suspensions of *V. ostreicida* were evaluated on lysosomal membrane stability (LMS) and bactericidal activity was evaluated as described above following (30). In all experiments, ASW at 31 ‰ salinity was utilized.

### 2.6 Data analysis

Embryotoxicity test data, representing the mean ± SD of 3 independent experiments, carried out in 6 replicate samples in 96-microwell plates, were analyzed by ANOVA plus Tukey’s post-test. EC_50_ values for embryotoxicity and LMS data were calculated at 95% confidence intervals (C.I.). Data on hemocyte and hemolymph functional parameters are the mean ± SD of at least 4 experiments, with each assay performed in triplicate. Statistical analyses were performed by the non-parametric Kruskal-Wallis test followed by Dunn’s test (p ≤ 0.05). All statistical calculations were performed using the GraphPad Prism version 7.03 for Windows, GraphPad Software, San Diego, CA, USA.

## 3 Results

### 3.1 Effects on early larval development

**Figure 1:**
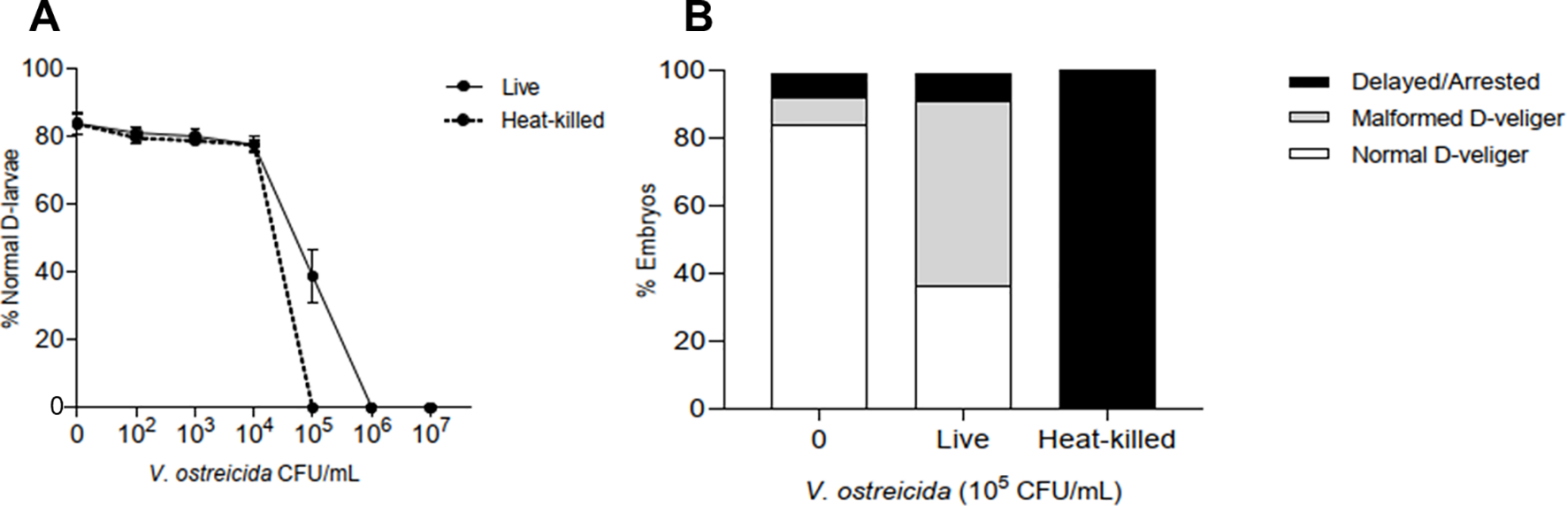
Effects of *V. ostreicida* on *M. galloprovincialis* embryo-larval development evaluated in the 48 h embryotoxicity assay. Fertilized eggs were exposed to different concentrations of live and heat-killed *V. ostreicida* suspension in ASW (from 10^2^ to 10^7^ CFU/mL). **(A)** Percentage of normal D-shaped larvae with respect to controls. **(B)** Percentage of larval phenotypes at 10*^5^* CFU/mL: normal D-veliger (white), malformed D-veliger (grey) and delayed/arrested larvae (black). Data represent the mean ± SD of 3 independent experiments carried out in 96-multiwell plates (6 replicates wells for each sample).

We first tested the potential pathogenicity of *V. ostreicida*, an oyster larval pathogen (25), on early larval development of *M. galloprovincialis*. Fertilized eggs were exposed to different concentrations (from 10^2^ to 10^7^ CFU/mL) of live and heat-killed *V. ostreicida* and the percentage of normal D-larvae was evaluated after 48 h post fertilization (hpf) (15, 30) (**Figure 1**). In controls, the percentage of normal D-larvae at 48 hpf was 84 ± 3%. The results, reported in **Figure 1A**, show that live *V. ostreicida* induced a concentration dependent decrease in normal development, with an EC_50_ = 9.64 x 10^3^ CFU/mL (95% C.I.: 8.04 – 11.60) (corresponding to a ratio bacteria:larvae of about 4:1). A strong effect was observed from concentrations > 10^5^ CFU/mL (-60% of normal D-larvae). Slightly lower EC_50_ values were obtained with heat-killed bacteria (3.09 x 10^3^ CFU/mL, 95% C.I.: 2.03 – 4.69), corresponding to a ratio bacteria:larvae of 1.24:1.

When the effects of live and heat-killed bacteria on larval phenotypes were compared at 10^5^ CFU/mL, the results indicate that live *V. ostreicida* mainly induced larval malformations (57% of total larvae), whereas challenge with heat-killed *V. ostreicida* resulted in 100% delayed or arrested larvae (**Figure 1B**).

### 3.2 *In vitro* effects of *V. ostreicida* on adult mussel hemocytes

#### 3.2.1 Effects of heat-killed bacteria

**Figure 2:**
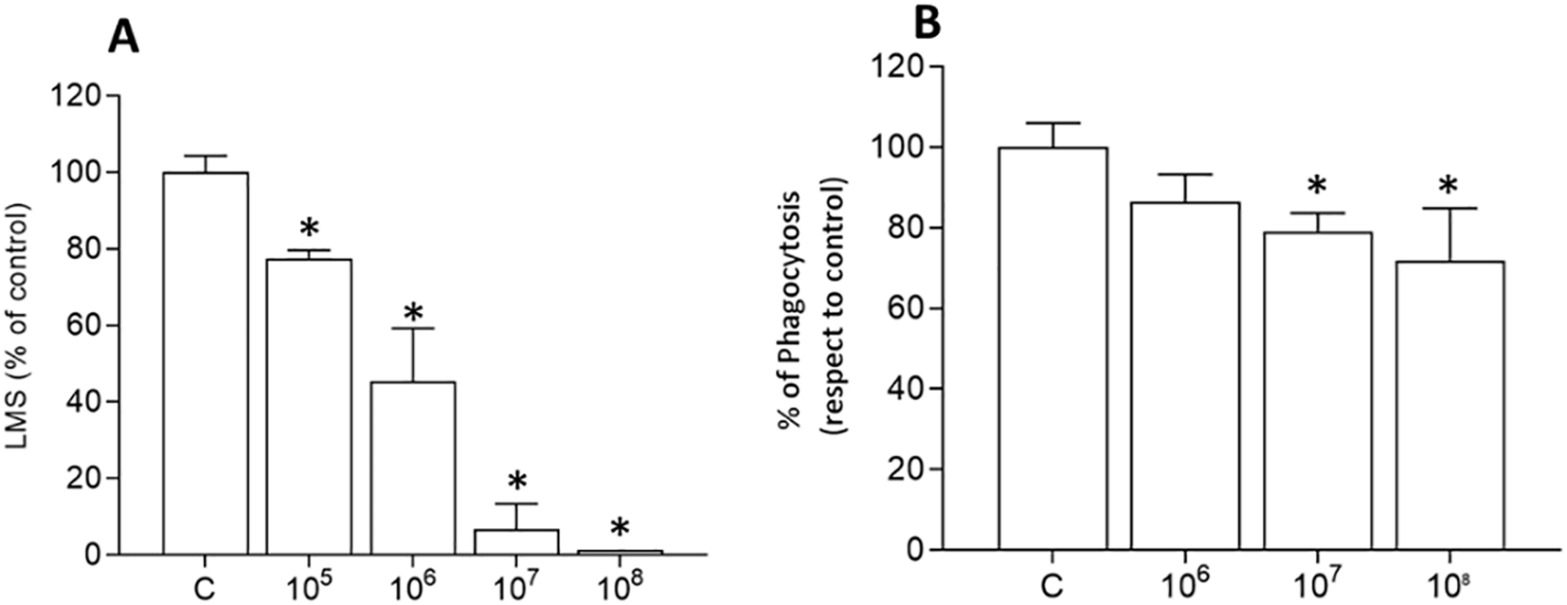
*In vitro* effects of heat-killed *V. ostreicida* on hemocyte lysosomal membrane stability (LMS) and phagocytic activity. **(A)** LMS: Hemocytes were exposed to different concentrations (from 10^5^ to 10^8^ CFU/mL) of heat-killed *V. ostreicida* suspensions in ASW for 30 min. **(B)** Phagocytosis: hemocytes were exposed to heat-killed *V. ostreicida* (from 10^6^ to 10^8^ CFU/mL) for 60 min. Data represent the mean ± SD of n=4 experiments. Statistical analyses were performed by non-parametric Kruskal-Wallis followed by Dunn’s multiple comparisons test, *p < 0.05.

**Figure 3:**
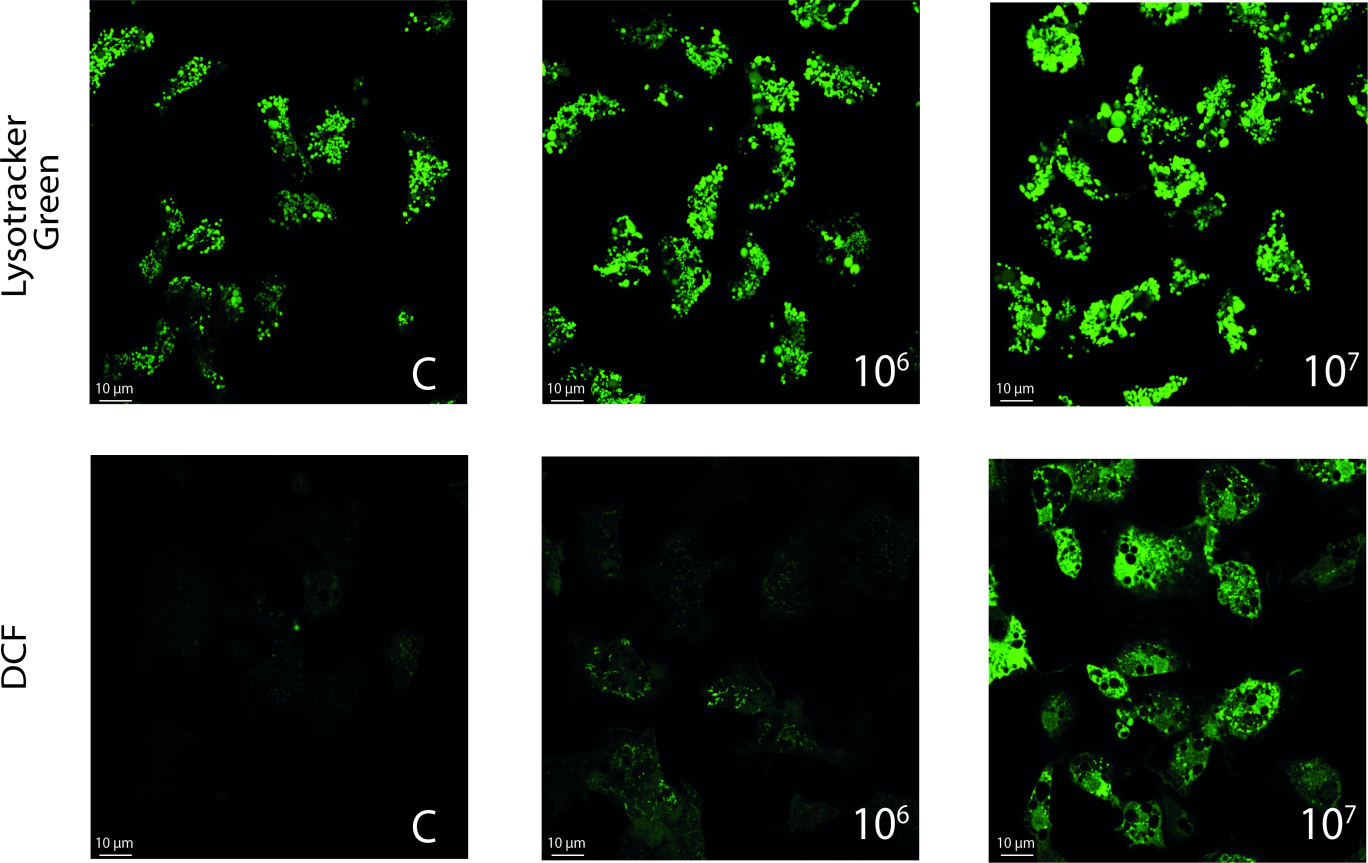
Confocal fluorescence microscopy: effects of *in vitro* exposure (30 min) of *Mytilus* hemocytes to heat-killed *V. ostreicida* suspensions at different concentrations (CFU/mL). Hemocytes from controls and challenged groups were loaded with LysoTracker Green (upper panel) and 2’,7’-Dichlorofluorescein DCF (lower panel). Representative images are reported (LysoTracker Green: ex: 504 nm, em: 511 nm; DCF: ex: 495 nm, em: 520 nm).

*In vitro* responses of hemocytes to heat-killed *V. ostreicida* suspensions in ASW were first evaluated, and data obtained for LMS and phagocytosis are reported in **Figure 2**. As shown in **Figure 2A**, a large and concentration dependent decrease in LMS was observed (from -20% with respect to controls at 10^5^ CFU/mL to complete destabilization at 10^7^ CFU/mL, **Figure 3A**), with an EC_50_ of about 10^6^ CFU/ml (7.88 x 10^5^ CFU/mL, 95% C.I.: 4.71 – 12.8). The hemocyte phagocytic activity was slightly reduced (about -20-25% respect with control) by exposure to the highest concentrations of bacteria, 10^7^ and 10^8^ CFU/mL (**Figure 2B**).

The effects of heat-killed bacteria on the lysosomal system were confirmed by CLSM (Confocal laser scanning microscopy) using LTG (Lysotracker green), a fluorescent dye that stains acidic compartments in live cells. As shown in **Figure 3** (upper panel) an increase in LTG fluorescence was progressively observed at increasing concentrations of bacteria, indicating lysosome enlargement and fusion events. In the same conditions, increased intracellular ROS production, evaluated by DCF fluorescence, was also observed (**Figure 3**, lower panel).

In contrast, no significant effects were observed in activation of extracellular immune parameters (ROS, NO production and lysozyme activity) at any time and bacteria concentration (**Supplementary Figure S1**).

#### 3.2.2 Effects of live bacteria

**Figure 4:**
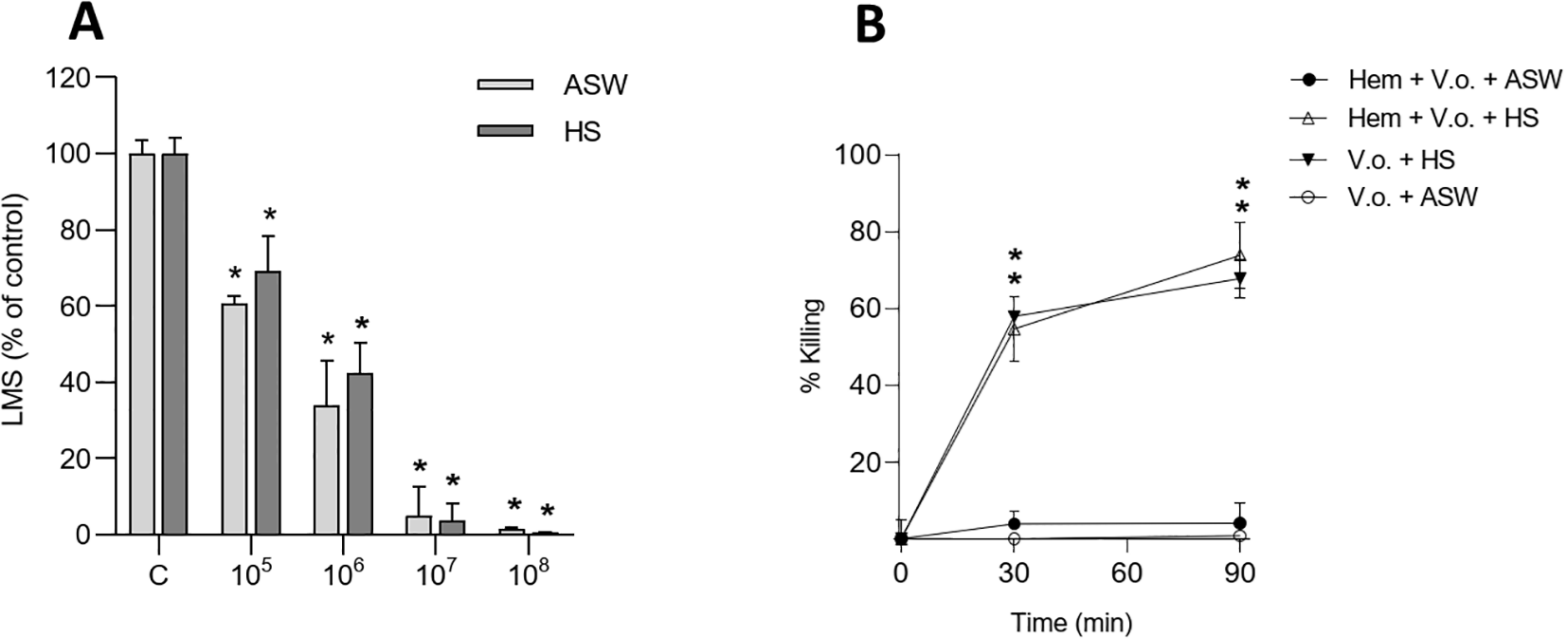
In *vitro* effects of live *V. ostreicida* in different media on hemocyte lysosomal membrane stability (LMS) and hemolymph bactericidal activity. **(A)** LMS: hemocyte monolayers were exposed to different concentrations (from 10^5^ to 10^8^ CFU/mL) of *V. ostreicida* resuspended in ASW or HS for 30 min. **(B)**actericidal activity: hemocyte monolayers were incubated for 30 and 90 min with *V. ostreicida* (5 x 10^6^ CFU/mL) resuspended in ASW (Hem+V.o.+ASW) or in HS (Hem+V.o.+HS). In parallel, *V. ostreicida* were incubated in ASW (V.o.+ASW) or HS (V.o.+HS) alone. At each time point, the number of viable, cultivable bacteria (CFU) was evaluated, and the results are expressed as percent values respect to time zero. Data represent the mean ± SD of n=4 experiments. Statistical analyses were performed by non-parametric Kruskal-Wallis followed by Dunn’s multiple comparisons test, *p < 0.05.

The *in vitro* effects of live *V. ostreicida* on mussel hemocytes were investigated in different media, ASW or HS, and the results are reported in **Figure 4**. As shown in **Figure 4A**, live *V. ostreicida* induced a similar dose-dependent decrease in LMS in both media, although with a slightly lower EC_50_ in ASW (2.23 x 10^5^ CFU/mL, 95% C.I.: 1.11 – 5.39), than in HS (6.52 x 10^5^ CFU/mL, 95% C.I.: 3.20 – 1.19). At the lowest concentration tested, the decrease in LMS induced by live *V. ostreicida* was larger (-40 and -30% with respect to controls in either ASW or HS) compared with that induced by heat-killed bacteria (see **Figure 2A**). However, complete lysosomal destabilization (>95%) was observed from 10^7^ CFU/mL.

The capacity of mussel hemocytes to kill *V. ostreicida* (5 x 10^6^ CFU/mL) was investigated using a bactericidal assay that evaluates the number of live, culturable bacteria at different times of incubation (30 and 90 min) (30). The bactericidal activity was measured in hemocytes in the presence of ASW or HS, as well as in HS and ASW alone, and data are expressed as the percentage of killed bacteria with respect to time zero (**Figure 4B**). The results showed the absence of killing by hemocytes in ASW. In contrast, a high bactericidal activity was due to HS, irrespectively of the presence of hemocytes (60% and 70% respectively, at 30 and 90 min).

**Figure 5:**
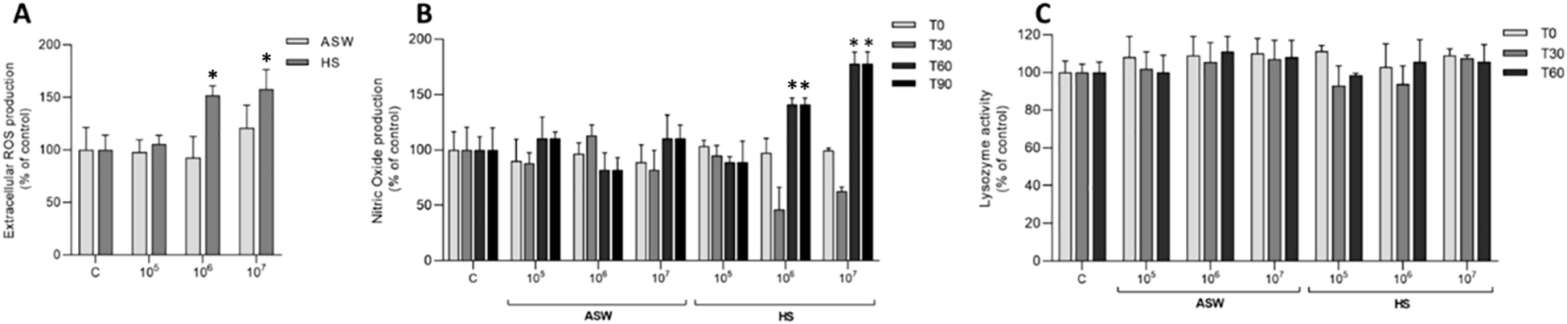
In *vitro* effects of live *V. ostreicida* on extracellular immune parameters of *M. galloprovincialis.* Extracellular ROS **(A)**, Nitric Oxide **(B)** production and lysozyme release **(C)**, were evaluated after incubation with *V. ostreicida* at 10^5^, 10^6^ and 10^7^ CFU/mL in ASW or HS (hemolymph serum). Data represent the mean ± SD of n=4 experiments. Statistical analyses were performed by non-parametric Kruskal-Wallis followed by Dunn’s multiple comparisons test, *p < 0.05.

Live bacteria induced a significant stimulation of extracellular ROS (**Figure 5A**) and NO production (**Figure 5B**), although only in HS and at the highest concentrations tested. No stimulation of lysozyme activity was observed in any experimental condition (**Figure 5C**).

### 3.3 *In vitro* effects of heat-killed and live *V. ostreicida* on adult oyster *M. gigas*

**Figure 6:**
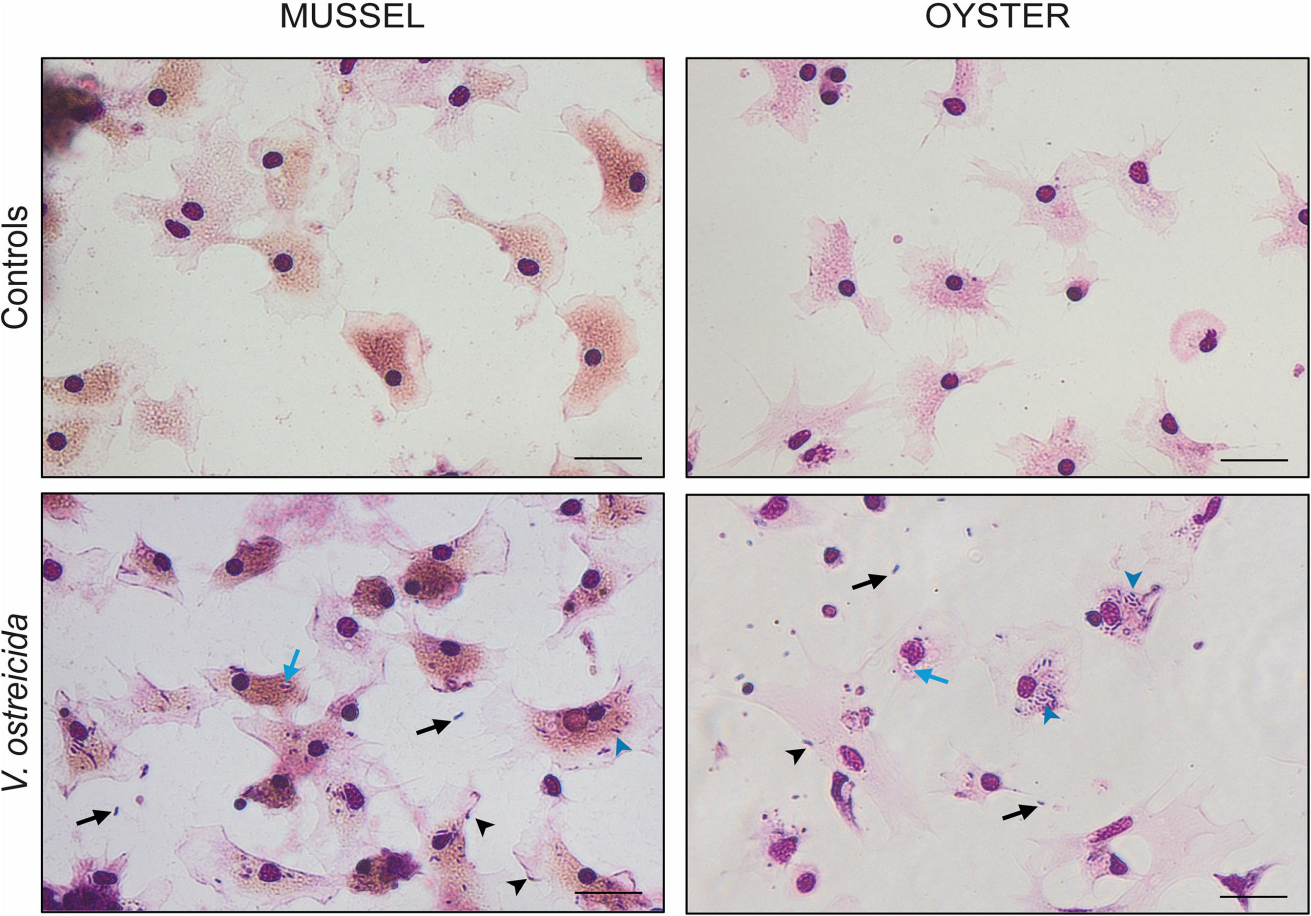
Giemsa staining of hemocytes of *M. galloprovincialis* and *M. gigas* (controls and incubated with live *V. ostreicida* suspensions in ASW (30 min, 10^6^ CFU/mL). Black arrows: bacteria in extracellular medium; black arrowheads: bacteria adherent to cell membranes; blue arrowheads: bacteria in cytoplasm; blue arrows: bacteria in vacuolar structures. Scale bar: 20 µm.

Representative images of Giemsa staining of mussel and oyster hemocytes exposed to live *V. ostreicida* (30 min, 10^6^ CFU/mL) are reported in **Figure 6**. Control hemocytes of both specimens were well spread on the support, showing different cytoplasmic extensions, with the nuclei colored in dark violet, the cytosol in pink, and intracellular vacuoles and lysosomes in pink-yellowish (much more evident in mussel hemocytes). In both mussel and oyster hemocytes challenged with *V. ostreicida*, bacteria were visible in the extracellular medium (black arrows), adherent to cell membranes (black arrow heads), as well within the cells, both in cytoplasm (blue arrowheads) and in vacuolar structures (blue arrows).

**Figure 7:**
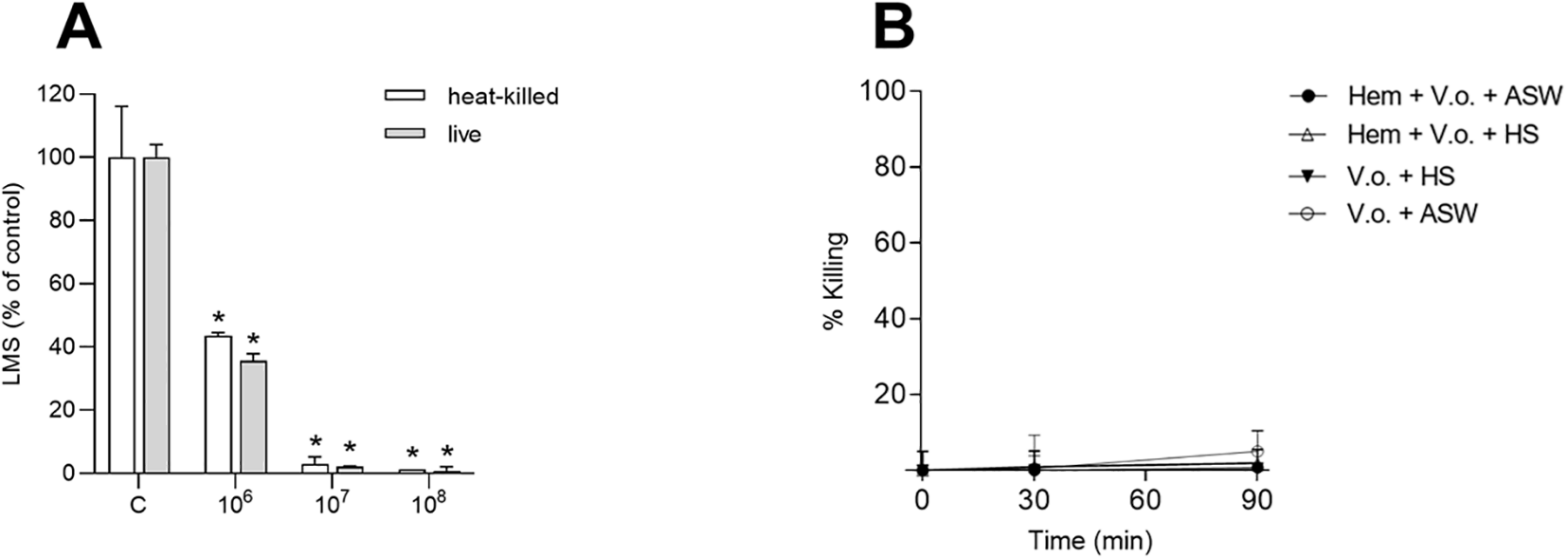
*In vitro* effects of *V. ostreicida* in *M. gigas* hemocytes. **(A)** LMS: hemocyte monolayers were exposed to different concentrations (from 10^6^ to 10^8^ CFU/mL) of heat-killed or live *V. ostreicida* suspensions for 30 min. **(B)** Bactericidal activity: hemocyte monolayers were incubated for 30 and 90 min with *V. ostreicida* (5 x 10^6^ CFU/mL) resuspended in ASW (Hem+V.o.+ASW) or in HS (Hem+V.o.+HS). In parallel, *V. ostreicida* were incubated in ASW (V.o.+ASW) or HS (V.o.+HS) alone. At each time point, the number of viable, cultivable bacteria (CFU) was evaluated, and the results are expressed as percent values respect to time zero. Data represent the mean ± SD of n=4 experiments. Statistical analyses were performed by non-parametric Kruskal-Wallis followed by Dunn’s multiple comparisons test, *p < 0.05.

When oyster hemocytes were incubated with *V. ostreicida* suspensions in ASW, a similar dose-dependent decrease in LMS was observed with both heat-killed and live bacteria (**Figure 7A**). At the lowest concentration tested (10^6^ CFU/mL), a decrease in LMS with respect to controls was observed (-60%), until almost complete destabilization (>95%) at 10^7^ CFU/mL. LMS EC_50_ values were comparable with those obtained in mussel hemocytes, although slightly lower for oyster hemocytes challenged with live suspension (2.6 x 10^5^ CFU/mL, 95% C.I.: 0.8 – 7.4), than with heat-killed bacteria (4.3 x 10^5^ CFU/mL, 95% C.I.: 2.60 – 7.6).

The capacity of oyster hemocytes to kill *V. ostreicida* (5 x 10^6^ CFU/mL) was evaluated in the bactericidal assay in the presence of ASW or HS, as well as in HS and ASW alone (**Figure 7B**). No bactericidal activity towards *V. ostreicida* was observed in any experimental condition (**Figure 7B**).

## 4 Discussion

The results of this work represent the first data on responses of *M. galloprovincialis* to *V. ostreicida*, a vibrio species previously identified as a larval oyster pathogen and associated with oyster mortalities in Spain (24, 25). Evidence is provided here of the effects of a *V. ostreicida* strain r172, isolated from a mortality episode of adult *M. galloprovincialis* in a shellfish production zone at the Ebro Delta River (27) on mussel larval development and hemolymph immune responses.

Since both mussel and oyster early developmental stages are likely to be more susceptible than adults to potential pathogens, not having fully developed the immune system (35, 36), we firstly tested the effects of *V. ostreicida* r172 in mussel early larvae. The results demonstrate that *V. ostreicida* affects normal *Mytilus* larval development, as shown by data from the 48 h embryo/larval assay. Heat-killed bacteria were also utilized in order to evaluate whether only live bacteria were able to interfere with larval development. Similar EC_50_ values, in the range of 10^3–^10^4^ CFU/mL, were obtained with both heat-killed and live bacteria. However, distinct effects on larval phenotypes were observed, with live bacteria mainly inducing shell malformations, and dead bacteria developmental arrest. The results indicate that normal development is affected by interactions of live *V. ostreicida* with mussel larvae, as previously observed with other bacterial isolated (15, 31). Moreover, the results obtained by heat-killed bacteria indicate that, products released by dead bacteria are involved in larval toxicity; these may include toxins, cell wall proteins, outer membrane proteins and lipopolysaccharides. Although the possible outcome of larval development at later stages cannot currently be predicted, the results indicate that *V. ostreicida* r172 is pathogenic to mussel early larvae. When compared with other live Vibrio tested so far in the same experimental conditions, the effect of *V. ostreicida* was comparable to those of other pathogenic bacterial species and strains: *V. bathipelagicus* induced larval death (14) and *V. coralliilyticus* and *V. tasmaniensis* LGP32 developmental arrest (14, 15).

*V. ostreicida* did not result in larval mortality; this is probably due to the low experimental ratio bacteria:larvae (about 4:1 at EC_50_ values for live bacteria). Although no data is available on concentrations of *V. ostreicida* in natural conditions in seawater, in this work, a wide range of concentrations were tested to fully cover also potentially natural environmental occurrence of *V. ostreicida*. In contrast, in oyster experimental trials focused on larval pathogenicity, much higher bacteria:larvae ratios were utilized (10^4^-10^5^/larva), possibly simulating the worst scenarios occurring in contaminated hatcheries. In 10–15 day-old larvae of the flat oyster *O. edulis*, the *V. ostreicida* type strain PP-203T at 10_5–_10_6_ CFU/mL induced 98% mortality from 72 to 96 h after inoculation (24, 25).

In adult mussels, the outcome of infection depends on the capacity of hemolymph components (hemocytes and hemolymph serum) to recognize and mount an effective defense response towards invading pathogens (17, 37). The microbicidal activity of bivalve hemolymph results from the combined action of the phagocytic process, carried out by hemocytes, with humoral defense factors such as agglutinins (e.g. lectins), lysosomal enzymes (e.g. lysozyme), toxic oxygen intermediates and various antimicrobial peptides (37). Therefore, immune responses to challenge with *V. ostreicida* were tested in *in vitro* short-term experiments on hemolymph samples. The results indicate that both heat-killed and live *V. ostreicida* suspensions induced a dose dependent lysosomal stress in hemocytes.

*In vitro* incubation with *V. ostreicida* may cause some loss of cell adherence from hemocytes monolayers in particular at high concentrations, that induced strong lysosomal damage. However, previous data (28) showed that in the same experimental conditions with other pathogenic heat-killed vibrios (*V.* sp*lendidus* LGP32 and *V. anguillarum*), loss in adherence could be observed only at longer incubation times (3 h) than those utilized in the present study, and could be quantified only by flow cytometry analysis of different hemocyte subpopulations.

Heat-killed bacteria decreased hemocyte LMS and phagocytic activity, at the same time stimulating intracellular ROS production, but no activation of extracellular immune defenses. The results suggest that some components of *V. ostreicida* may interfere with hemocyte membranes and intracellular processes involved in immune responses.

When considering all data obtained with heat-killed *V. ostreicida*, experiments were performed as previously described using a whole (unwashed) bacterial suspension (28). Therefore, it must be underlined that the observed effects may be due to products that are not naturally released by bacteria, as well as to bacterial components released from bacteria killed by hemolymph components or by the larvae, or both. However, the results indicate that heat-killed *V. ostreicida* induced responses in mussel and oyster hemocytes similar to those elicited by the bivalve pathogen *V.* sp*lendidus* in the same experimental conditions (28).

Live bacteria induced a similar decrease in LMS, independent of the presence of soluble serum components; however, stimulation of extracellular ROS and NO production was observed, although only in the presence of HS, and at higher bacterial concentrations, indicating some role for soluble hemolymph components in recognition of bacteria by hemocytes and subsequent, although poor, immune activation. Accordingly, when hemocytes were incubated with live bacteria in ASW they were unable to kill *V. ostreicida*, suggesting that observed bactericidal activity was essentially due to hemolymph serum.

The results obtained with *M. gigas* indicate a similar effect of heat-killed and live bacteria on hemocyte LMS, confirming lysosomal stress. However, no activation of extracellular defenses (ROS, NO and lysozyme release) were observed in any condition (not shown). Moreover, in the oyster, neither hemocytes alone nor HS showed the capacity to kill *V. ostreicida*. The results indicate the presence in the hemolymph of *M. galloprovincialis*, but not in that of *M. gigas*, of specific soluble hemolymph components that are responsible for the direct bactericidal activity observed towards *V. ostreicida*. These may involve specific antimicrobial peptides or other factors specifically directed towards *V. ostreicida* whose identity needs further investigation.

This data further underlines the differences in the role of hemolymph components in the immune responses of mussels and oysters to potential invading pathogens (17). As recently reviewed in (38), advances in molecular immunobiology provided deeper insights into the cellular and molecular mechanisms underlying the robust defense system of *M. galloprovincialis*, which allows this species to efficiently cope with a broad range of infections. *M. galloprovincialis* is one of the species with the highest amount of different families of antimicrobial peptides (AMPs) in the animal kingdom. Moreover, the complexity of the immune repertoire is further enhanced by the presence in mussel genome of extensive hemizygous regions, which exhibit extensive presence/absence variation (PAV) among individuals, and affects all known AMP families to varying degrees (38). In this light, studies on the transcriptomic responses of different bivalve species can help unraveling the mechanisms underlying resilience to different pathogenic infections (39). In this work, the direct bactericidal activity towards *V. ostreicida* observed by the soluble hemolymph fraction of *M. galloprovincialis*, but not if *M. gigas*, suggests the presence in the Mediterranean mussel of constitutive plasma peptides/proteins specifically directed to this vibrio species. However, only proteomic/peptidomic studies can identify differences in the presence of hemolymph plasma of immune effectors between mussels and other bivalve species (40, 41).

Further experiments of *in vivo* challenge of mussels with *V. ostreicida* will provide information on its virulence, time course of infection and hemolymph immune responses. These data will help elucidating the capacity of *M. galloprovincialis* to cope with this bacterial infection. The results of these studies will contribute to increasing knowledge on the interactions between potentially pathogenic vibrios and the bivalve host, thus potentially predicting causes of mortalities which are increasingly affecting aquaculture farms worldwide causing significant ecological and economic losses.

## Data Availability

The raw data supporting the conclusions of this article will be made available by the authors, without undue reservation.
