# Supplementary material for: Interactions between the oyster larvae pathogen Vibrio ostreicida and the bivalve hosts Mytilus galloprovincialis and Magallana gigas
Source: Front Immunol. 2025 Nov 26;16:1711477. doi: 10.3389/fimmu.2025.1711477 (PMC12689417; doi:10.3389/fimmu.2025.1711477)
Supplement: Supplementary file 1 [file DataSheet1.docx]

Supplementary Material to

**Interactions between the oyster larvae pathogen *Vibrio ostreicida* and the bivalve hosts *Mytilus galloprovincialis* and *Magallana gigas*.**

Martina Leonessi^1,2*^, Manon Auguste^1,2^, Jose R. Lopez^3,4^, Teresa Balbi^1,2^, Caterina Ciacci^5^, Caterina Oliveri^1^, Luigi Vezzulli^1,2^, Dolors Furones^3^, Laura Canesi^1,2^

^1^Department of Earth, Environmental and Life Sciences (DISTAV), University of Genoa, Genoa 16132, Italy

^2^National Biodiversity Future Center, Palermo, Italy

^3^IRTA, Aquaculture Program. La Ràpita, Spain

^4^Universidade de Santiago de Compostela, Santiago de Compostela, Spain

^5^Department of Biomolecular Sciences (DIBS), University ‘Carlo Bo’ of Urbino, Urbino, Italy

# Materials and Methods

# Larval assays

# All procedures were carried out following the protocol described in (1). For each exposure condition experiments were carried out utilizing three different single pairings. Six replicates were made for each treatment, in 96 wells, each containing about 50 embryos. A total of 300 larvae were thus examined for each condition, according to the protocol ISO (2). Observations were acquired manually by an operator blind to the experimental conditions.

**Evaluation of functional hemocyte parameters**

LMS was evaluated in hemocyte monolayers of *M. gigas* and *M. galloprovincialis* by the Neutral Red Retention Time (NRRT) assay (3) as previously described (4–6). Hemocyte monolayers were pre-incubated for 30 min with 20 µL of different *V. ostreicida* suspensions, either live (resuspended in ASW or HS) or heat-killed (in ASW), at different concentrations (from 10^8^ to 10^5^ CFU/mL). Samples were washed out and incubated with a neutral red (NR) (Sigma-Aldrich, Milan, Italy) solution: 20 µg/mL for 7 min and 40 µg/mL (in ASW) for 15 min for respectively oyster and mussel monolayers. At the end of incubation, excess dye removed, a drop of ASW was added and the slides were observed under an optical microscope from time zero every 15 min. The percentage of cells showing loss of dye from lysosomes in each field (10 fields each containing 8-10 cells) was evaluated until 50% of the cells showed sign of lysosomal leaking. All incubations were carried out at 18 °C.

Phagocytic activity was evaluated by the uptake of NR-stained zymosan by mussel hemocyte monolayers. Briefly, samples were incubated first with ASW (control) or heat-killed *V. ostreicida* suspensions (from 10^5^ to 10^8^ CFU/mL) for 1 h and then with NR-stained zymosan in 0.05 M Tris-HCl buffer, pH 7.6 (TBS) for 1 h. Then monolayers were washed three times with ASW, fixed with Baker’s calcium formol, and mounted in Kaiser’s glycerol gelatine medium for microscopical examination with a fluorescent microscope. For each slide, the percentage of phagocytic hemocytes was calculated from minimum of 200 cells in triplicate samples (6,7).

Lysozyme activity, ROS and NO production were measured as previously described (6). Aliquots of whole hemolymph (500 µL) were incubated with either live and heat-killed suspension of *V. ostreicida* (final concentrations 10^5^, 10^6^ and 10^7^ CFU/mL) at 18 °C for different times depending on the endpoint measured. Lysozyme activity was evaluated spectrophotometrically at 450 nm using a suspension of *Micrococcus lysodeikticus* (15 mg/100 mL in 66 mM phosphate buffer, pH 6.4) after incubation with bacteria for 0, 30 and 60 minutes. Nitric oxide (NO) production was evaluated by Griess reaction after incubation with bacteria for 0, 30, 60 and 90 minutes; samples were centrifuged (12000 x g for 30 min at 4 °C) and the supernatants were incubated for 10 min in the dark with 1% (w/v) sulphanilamide in 5% H_3_PO_4_ and 0.1% (w/v) N-(1-naphthyl)-ethylenediamine dihydrochloride. Samples were read at 540 nm and the molar concentration of NO_2_^-^ in the samples were calculated from standard curves generated using known concentrations of sodium nitrite. Extracellular ROS generation was measured by the reduction of cytochrome c. Hemolymph samples were incubated for 30 min with a cytochrome c solution (75 µM ferricytochrome c in TBS). Samples were read at 550 nm. Cytochrome c in TBS was utilized as a blank. The results were expressed as changes in OD per mg protein. Protein content was determined using the Bradford method using bovine serum albumin (BSA) as standard. All data are reported per mg/protein sample and expressed a percentage of control values.

# Results


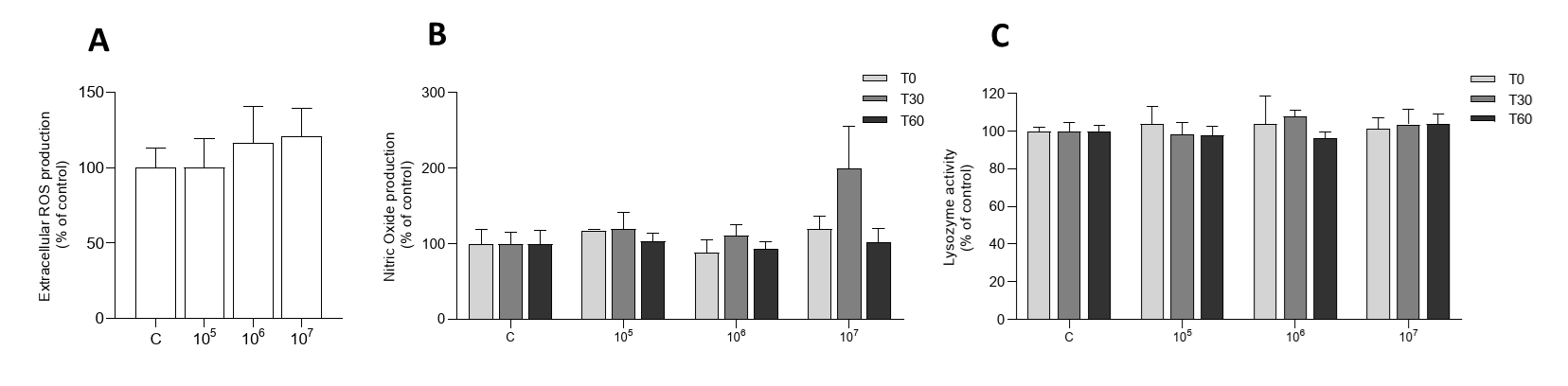


Supplementary Figure 1. *In vitro* effects of heat-killed *V. ostreicida* on extracellular immune parameters of *M. galloprovincialis*. Extracellular ROS (A), Nitric Oxide (B) production and lysozyme release (C), were evaluated after incubation with *V. ostreicida* at 10⁵, 10⁶ and 10⁷ CFU/mL in ASW. Data represent the mean ±SD of n=4 experiments. Statistical analyses were performed by non-parametric Kruskal-Wallis followed by Dunn’s multiple comparisons test. No significant differences were observed among different groups (p > 0.05).

# References

1. Fabbri R, Montagna M, Balbi T, Raffo E, Palumbo F, Canesi L. Adaptation of the bivalve embryotoxicity assay for the high throughput screening of emerging contaminants in Mytilus galloprovincialis. Mar Environ Res. 2014 Aug;99:1–8.

2. International Standards Organization. Water quality — Determination of the toxicity of water samples on the embryo-larval development of Japanese oyster (Crassostrea gigas) and mussel (Mytilus edulis or Mytilus galloprovincialis). 2015; Available from: ISO:17244:2015

3. OSPAR Commission. Background documents and technical annexes for biological eﬀects monitoring. In 2013. p. 239. Available from: www.ospar.org.

4. Lasa A, Auguste M, Lema A, Oliveri C, Borello A, Taviani E, et al. A deep‐sea bacterium related to coastal marine pathogens. Environ Microbiol. 2021 Sep;23(9):5349–63.

5. Auguste M, Rahman FU, Balbi T, Leonessi M, Oliveri C, Bellese G, et al. Responses of Mytilus galloprovincialis to challenge with environmental isolates of the potential emerging pathogen Malaciobacter marinus. Fish Shellfish Immunol. 2022 Dec;131:1–9.

6. Auguste M, Leonessi M, Balbi T, Doni L, Oliveri C, Vezzulli L, et al. Seasonal fluctuations of hemolymph microbiota and immune parameters in Mytilus galloprovincialis farmed at La Spezia, Italy. Aquaculture. 2024 Jan;578:740028.

7. Balbi T, Cortese K, Ciacci C, Bellese G, Vezzulli L, Pruzzo C, et al. Autophagic processes in Mytilus galloprovincialis hemocytes: Effects of Vibrio tapetis. Fish Shellfish Immunol. 2018 Feb;73:66–74.
